# Comparative Transcriptome Profiling Reveals Two WRKY Transcription Factors Positively Regulating Polysaccharide Biosynthesis in *Polygonatum cyrtonema*

**DOI:** 10.3390/ijms241612943

**Published:** 2023-08-18

**Authors:** Wu Jiang, Jiadong Chen, Xiaojing Duan, Yaping Li, Zhengming Tao

**Affiliations:** 1 Zhejiang Institute of Subtropical Crops, Zhejiang Academy of Agricultural Sciences, Wenzhou 325005, China; jiangwu@zaas.ac.cn (W.J.); chenjd@zaas.ac.cn (J.C.); duanxj@zaas.ac.cn (X.D.); 2 Zhejiang Provincial Key Laboratory of Resources Protection and Innovation of Traditional Chinese Medicine, Zhejiang A&F University, Hangzhou 311300, China; liyaya425429@gmail.com

**Keywords:** polysaccharide biosynthesis, regulatory mechanism, germplasms, transcriptome, *Polygonatum cyrtonema*

## Abstract

*Polygonatum cyrtonema* (*P. cyrtonema*) is a valuable rhizome-propagating traditional Chinese medical herb. Polysaccharides (PCPs) are the major bioactive constituents in *P. cyrtonema*. However, the molecular basis of PCP biosynthesis in *P. cyrtonema* remains unknown. In this study, we measured the PCP contents of 11 wild *P. cyrtonema* germplasms. The results showed that PCP content was the highest in Lishui Qingyuan (LSQY, 11.84%) and the lowest in Hangzhou Lin’an (HZLA, 7.18%). We next analyzed the transcriptome profiles of LSQY and HZLA. Through a qRT-PCR analysis of five differential expression genes from the PCP biosynthesis pathway, phosphomannomutase, UDP-glucose 4-epimerase (galE), and GDP-mannose 4,6-dehydratase were determined as the key enzymes. A protein of a key gene, *galE1*, was localized in the chloroplast. The PCP content in the transiently overexpressed *galE1* tobacco leaves was higher than in the wild type. Moreover, luciferase and Y1H assays indicated that PcWRKY31 and PcWRKY34 could activate *galE1* by binding to its promoter. Our research uncovers the novel regulatory mechanism of PCP biosynthesis in *P. cyrtonema* and is critical to molecular-assisted breeding.

## 1. Introduction

*Polygonati rhizoma* (known as “Huangjing” in Chinese, the rhizome of several *Polygonatum* species in Liliaceae family) is a noted traditional Chinese herbal medicine that has been extensively used over thousands of years [1]. *Polygonati rhizoma* is prescribed as dried rhizomes from three legal sources in the Pharmacopeia of China, including *Polygonatum sibiricum*, *P. kingianum,* and *P. cyrtonema* [2]. Among the three legal species of Huangjing, *P. cyrtonema* is one of the most cultivated plants for its optimal medicinal quality and yield. Currently, *P. cyrtonema* is used for pharmaceutical ingredients based on TCM theory and is also widely used in dietary supplements and nutraceutical agents.

Much research has been conducted on identifying *P. cyrtonema* chemical constituents, mainly consisting of polysaccharides (PCPs), saponins, alkaloids, and various flavonoids [3]. Currently, the high PCP content in *P. cyrtonema* is highly praised in the Chinese herbal medicine market [4]. The identification of numerous PCPs with medicinal properties, including antiviral [5,6], antioxidant [7,8,9], antitumoral [10], and immunomodulatory activities [11,12,13,14], has sparked significant interest in the field of pharmaceutical research and development. PCPs typically exhibit characteristics, such as a large molecular weight (MW) and a repetitive structure, which are often not found in conventional small molecule pharmaceuticals [15]. Specifically, the pharmacodynamics of a given PCP can be attuned by adjusting its molecular mass and structural features [16,17]. The distinctive qualities of PCPs, such as their eco-friendliness, water solubility, and minimal toxicity, make them well suited as raw materials in the pharmaceutical industry [15,18,19,20,21,22]. In addition, the highly cross-linked networks of PCPs found in cell walls play a significant role in various crucial physiological processes, including growth [23,24], stress protection [25,26], and signal transduction [27,28,29].

Several studies have confirmed that the process of synthesizing plant PCPs involves the participation of several enzymes, including β-fructofuranosidase (sacA), hexokinase (HK), fructokinase (FRK), and phosphoglucomutase (PGM). These enzymes are responsible for the biosynthesis of nucleoside diphosphate sugars (NDP-sugars) [30,31,32,33,34,35,36]. These activated NDP-sugar precursors are then added to PCP residues in high amounts, promoting the formation of plant PCPs through a series of reactions catalyzed by glycosyltransferases (GT) [37,38].

Earlier research has indicated that R2R3-MYB genes, including *AtMYB5* and *AtMYB61*, play a vital role in the synthesis of seed mucilage. The Arabidopsis *myb61* mutant exhibited a notable decrease in PCP levels within its seed mucilage [39]. These results indicate that R2R3-MYB members play roles in the biosynthesis of plant PCPs. It was demonstrated that DoHY5 directly binds the promoters of DoGMPP2 and DoPMT28 (involved in PCP biosynthesis) to activate their expression and promote PCP biosynthesis [40]. MYB58/63 proteins have been implicated in the biosynthesis of cell wall PCPs in grass species. For instance, in rice, OsMYB58/63, along with AtMYB63 in Arabidopsis, has been found to activate the expression of the secondary cell wall-specific cellulose synthase gene OsCesA7 in rice protoplasts. [41]. MYB58/63 proteins have the ability to stimulate the biosynthesis of both lignin and cell wall PCPs in grass species [42]. Overexpressing *SbMYB60* has a positive impact on the expression of genes involved in PCP biosynthesis [43]. Previous studies on *P. cyrtonema* mainly focus on cultivation techniques, pharmacology function, processing methods, and chemical constituents. However, there are no reports on the enzymes and genetic information of genes responsible for biosynthesis and PCP metabolic pathways in *P. cyrtonema*.

Here, we measured the PCP contents of 11 wild *P. cyrtonema* germplasms which cultivated in Zhejiang Province, China. RNA sequencing of rhizomes from two germplasms with the largest contrast in PCP content was performed to study the transcriptomics, analyze PCP synthesis pathways, and explore and annotate genes related to PCP biosynthesis. We further identified key genes involved in PCP synthesis in *P. cyrtonema* by differential expression genes analysis and real-time qPCR validation. We verified the subcellular localization and function of the key gene in tobacco leaves. Moreover, the transcriptional modulators of key genes in the PCP biosynthesis pathway were identified by the dual-luciferase assay and the yeast one-hybrid method. The PCP biosynthesis pathway and key genes were first identified in this study. These results laid a foundation for enriching the gene resources and regulatory mechanism of PCP synthesis pathways in *P. cyrtonema*. Our results provide reference data for identifying and discovering key genes involved in medical plants’ biosynthesis of active ingredients. This study will help guide us in conducting future breeding programs.

## 2. Results

### 2.1. Total PCP Content of Various P. cyrtonema Germplasms

We collected 11 *P. cyrtonema* germplasms widely cultivated in Zhejiang Province, China. We analyzed the PCP contents of dried *P. cyrtonema* rhizomes from different germplasms. Among the germplasms, LSQY exhibited the highest PCP content at 11.84%. WZTS and JHPA closely followed with PCP contents of 9.08% and 9.58%, respectively. Conversely, HZLA had the lowest PCP content at 7.18%. WZRA and LSLQ also showed relatively lower PCP contents of 7.35% and 9.74%, respectively (Table 1). Due to the substantial contrast in PCP content between LSQY and HZLA, we chose these two germplasms for the construction of transcriptomic libraries. The main objective was to identify key genes associated with PCP biosynthesis.

### 2.2. Illumina Sequencing, Assembly and Unigene Annotation

**Figure 1:**
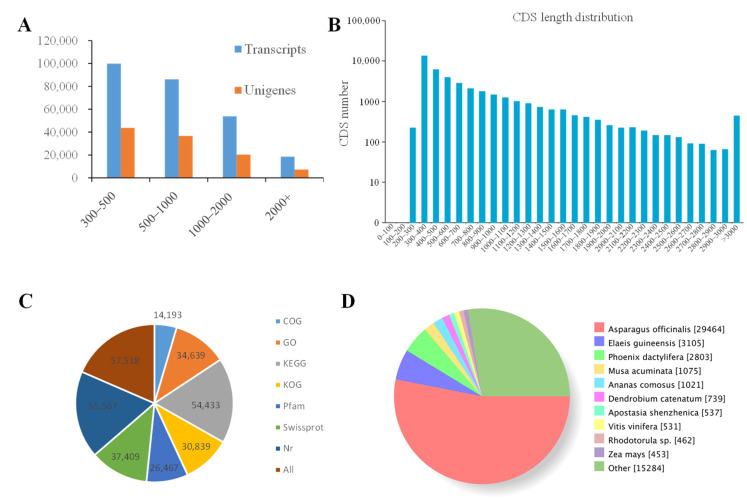
Characteristics of unigenes obtained from Illumina sequencing. (**A**) The length distribution of transcripts and unigenes in *Polygonatum cyrtonema*. (**B**) The length distribution of all CDS. (**C**) The annotation of unigenes based on different databases. (**D**) Diagram of *Polygonatum cyrtonema* homologous genes identified in other plants.

In total, we generated 4514 Gb of sequence data consisting of 150,464,765 short reads from six cDNA libraries (Table S1). In total, 5,422,742 transcripts with a mean length of 877.65 bp and 108,195 unigenes with a mean length of 855.15 bp were generated by Trinity software (v2.0.13). We obtained 99,828 transcripts and 43,728 unigenes with lengths <500 bp, 86,132 transcripts and 36,785 unigenes with lengths from 500 to 1000 bp, 53,792 transcripts and 20,341 unigenes with lengths from 1000 to 2000 bp, and 18,620 transcripts and 7341 unigenes with lengths >2000 bp (Figure 1A). Figure 1B shows the length distribution of the CDS. To annotate unigenes, all unigene sequences of *P. cyrtonema* were searched against protein databases. These protein databases include the COG (annotated 14,193 unigenes), GO (annotated 34,639 unigenes), KEGG (annotated 54,433 unigenes) KOG (annotated 30,839 unigenes), Pfam (annotated 26,467 unigenes), Swiss-Prot (annotated 37,409 unigenes), and Nr databases (annotated 55,567 unigenes). A total of 57,518 were annotated by at least one database (Figure 1C). The top five largest group of *P. cyrtonema* homologous genes were identified in *Asparaus officinalis* (29,464 genes), *Elaeis guineensis* (3105 genes), *Phoenix dactylifera* (2803 genes), *Musa acuinata* (1075 genes), and *Ananas comosus* (1021 genes) (Figure 1D).

### 2.3. Classification of KOG, GO, and KEGG Terms

The integrity of *P. cyrtonema* transcriptome and the accuracy of unigenes annotation were evaluated by the KOG database. A total of 30,839 unigenes were clustered into 25 functional categories (Figure S1A). The largest category was “general function prediction only” (8609 genes), followed by “post-translational modification, protein turnover, chaperones” (2525 genes), and “signal transduction mechanisms” (2385 genes) (Figure S1A). A total of 34,639 were assigned to GO terms. Within the cellular component category, the largest GO term was “cell” (26,895 genes), the second largest was “cell part” (26,846 genes), and the third was “organelle” (20,230 genes). Within the molecular function category, “binding” (20,623 genes), “antioxidant activity” (18,398), and “structural molecule activity” (2357 genes) were the three most abundant terms (Figure S1B). Within the biological process category, the three largest GO terms were “cellular process” (24,475 genes), “metabolic process” (22,111 genes), and “biological regulation” (24,475 genes). For the KEGG pathway analysis, the KEGG database blast against annotated unigenes and 17,094 unigenes was mapped onto 131 KEGG pathways (Table S2). Furthermore, 3202 unigenes were clustered into the PCP biosynthesis-related pathways (Table S3).

### 2.4. Identification of DEGs between Different Samples

**Figure 2:**
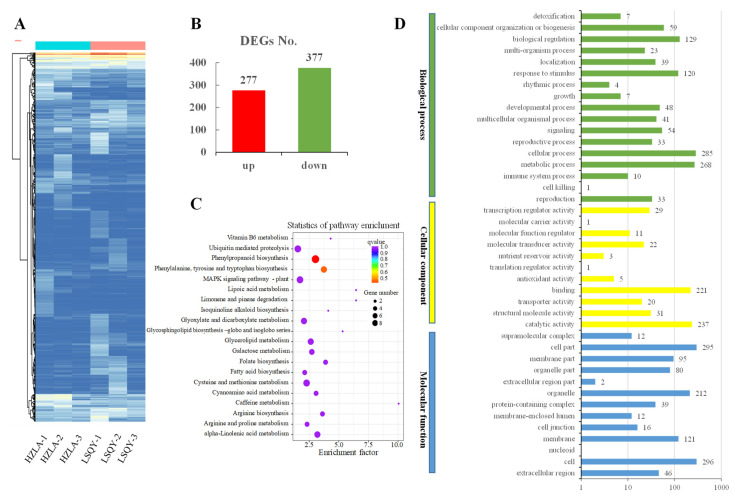
Identification of DEGs between LSQY and HZLA. (**A**) Cluster diagram of differential gene expression. (**B**) The up-regulated and down-regulated DEG number. (**C**) KEGG enrichment analysis of the DEGs’ variation between LSQY and HZLA samples. The 20 pathways with the most significant enrichment are shown. (**D**) GO enrichment analysis of the DEGs.

**Figure 3:**
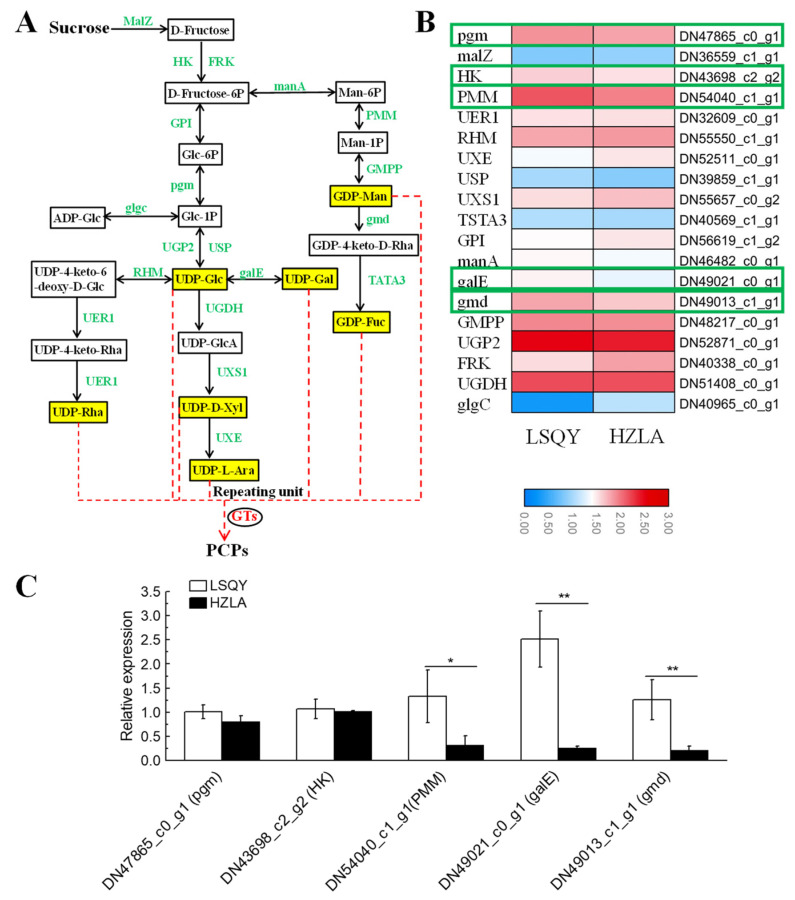
Identification and expression validation of key genes involved in PCP biosynthesis. (**A**) Proposed PCP biosynthesis pathways in Polygonatum cyrtonema. Activated monosaccharide units were marked in yellow. Abbreviations: MalZ: alpha-glucosidase; HK: hexokinase; FRK: fructokinase; GPI: glucose-6-phosphate isomerase; manA: mannose-6-phosphate isomerase; pgm: phosphoglucomutase; PMM: phosphomannomutase; GMPP: mannose-1-phosphate guanylyltransferase; UGP2: UTP-glucose-1-phosphate uridylyltransferase; glgC: glucose-1-phosphate adenylyltransferase; USP: UDP-sugar pyrophosphorylase; RHM: UDP-glucose 4,6-dehydratase; galE: UDP-glucose 4-epimerase; gmd: GDP-mannose 4,6-dehydratase; TSTA3: GDP-L-fucose synthase; UER1: 3,5-epimerase/4-reductase; UGDH: UDP-glucose 6-dehydrogenase; UXS1: UDP-glucuronate decarboxylase; UXE: UDP-arabinose 4-epimerase; GTs: glycosyltransferases. (**B**) Comparison of gene expression level between LSQY and HZLA using a logarithm of FPKM values. (**C**) Validation of the upregulated genes in LSQY from RNA-seq data using qRT-PCR. Statistically significant differences are indicated (* *p* < 0.05, ** *p* < 0.01). Data are means ± SD of three biological replicates.

The expression pattern of DEGs between the LSQY and HZLA samples is presented by a heatmap (Figure 2A). In detail, a total of 654 DEGs were found, and there were 277 LSQY highly expressed unigenes (Figure 2B; Table S4). Figure 3C shows 20 enriched KEGG pathways. The top three enriched KEGG pathways were “phenylpropanoid biosynthesis”, “Phenylalanine, tyrosine and tryptophan biosynthesis”, and “plant hormone signal transduction” (Figure 2C). GO term analysis of the DEGs identified 41 GO terms. The largest biological process category in GO terms was the “cellular process” (285 genes), “metabolic process” (268 genes), and “biological regulation” terms (129 genes) (Figure 2D). The largest molecular function categories in the GO terms were the “catalytic activity” (237 genes), “binding” (221 genes), and “structural molecule activity catalytic activity” terms (31 genes). Within the cellular component, “cell” (296 genes), “cell part” (296 genes), and “organelle” (212 genes) were the three most abundant terms (Figure 2D).

### 2.5. Identification of DEGs and Key Genes Involved in PCP Biosynthesis

To identify potential genes involved in PCP biosynthesis, we annotated the transcripts associated with the map00500, map00520, and map00051 pathways (Figure 3A). We identified 20 kinds of enzyme-encoding genes related to PCP biosynthesis. Among these, the genes involved in synthesizing UDP-glucose, UDP-D-xylose, and GDP-mannose showed a high expression level. We identified eight important DEGs in PCP biosynthesis, including five associated with starch and sucrose metabolism and three related to amino and nucleotide sugar metabolism. We also identified the PCP biosynthesis pathway genes from RNA-seq expression profiles; of them, there were five DEGs, including the genes encoding hexokinase (*HK1*), phosphomannomutase (*PMM1*), phosphoglucomutase (*pgm1*), GDP-mannose 4,6-dehydratase (*gmd1*), and UDP-glucose 4-epimerase (*galE1*) (Figure 3B). Their expression was further verified by qRT-PCR. We found that the *galE1* expression was significantly different between LSQY and HZLA, followed by *PMM1* and *gmd1*, suggesting that they are key genes contributing to the difference in PCP biosynthesis between LSQY and HZLA (Figure 3C). Afterward, we successfully cloned the CDS and promotor sequence of *PcgalE1* and used them for further analysis.

### 2.6. Subcellular Localization and Functional Verification of PcgalE1

**Figure 4:**
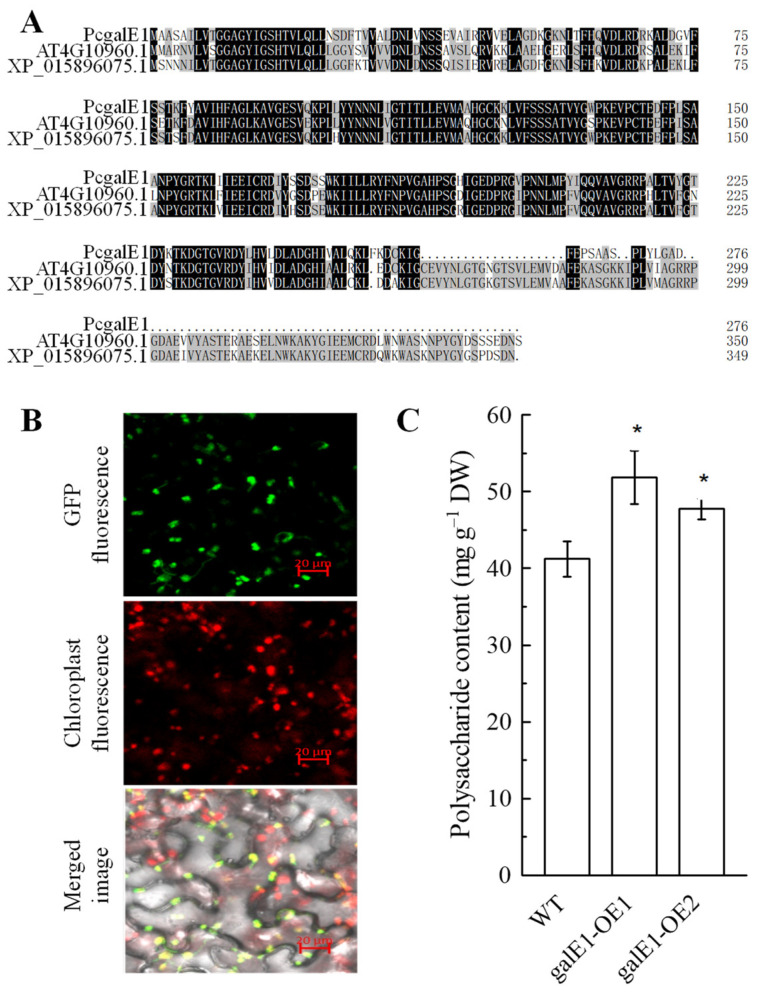
Sequence analysis, subcellular location, and functional verification of PcgalE1. (**A**) Alignment of the amino acid sequence of PcgalE1 with its homologue from Arabidopsis (AT4G10960.1) and Ziziphus jujube (XP_015896075.1). (**B**) Subcellular localization of PcgalE1. PcgalE1-GFP fusion plasmid was transiently expressed in tobacco leaves and co-localized with the chloroplast autofluorescence. Scale bars = 20 μm. (**C**) Polysaccharide content in the wild type and PcgalE1 transiently overexpressed N. benthamiana lines. DW, dry weight. Data are means ± SD of five biological replicates. Asterisks indicate significant differences at *p* < 0.05.

The multiple sequence alignment result showed that the protein sequence of PcgalE1 in *P. cyrtonema* exhibits a high degree of similarity to its homologs in *Arabidopsis* and *Ziziphus jujuba* (Figure 4A). Subcellular localization results showed that the GFP signals of PcgalE1-GFP were co-localized with the autofluorescence of chloroplast, indicating that PcgalE1 was localized to chloroplast (Figure 4B).

To further verify the function of PcgalE1 in PCP biosynthesis, *PcgalE1* was transiently expressed in tobacco leaves. PCP contents were detected in wild-type and *PcgalE1* transiently expressed leaves. Our results demonstrated that PCP content was significantly higher in *PcgalE1* overexpressed tobacco leaves than in wild-type leaves (Figure 4C). Our findings revealed that *PcgalE1* plays a key role in PCP biosynthesis and accumulation.

### 2.7. Screening and Identification of Transcriptional Modulators Involved in the PCP Biosynthesis Pathway

**Figure 5:**
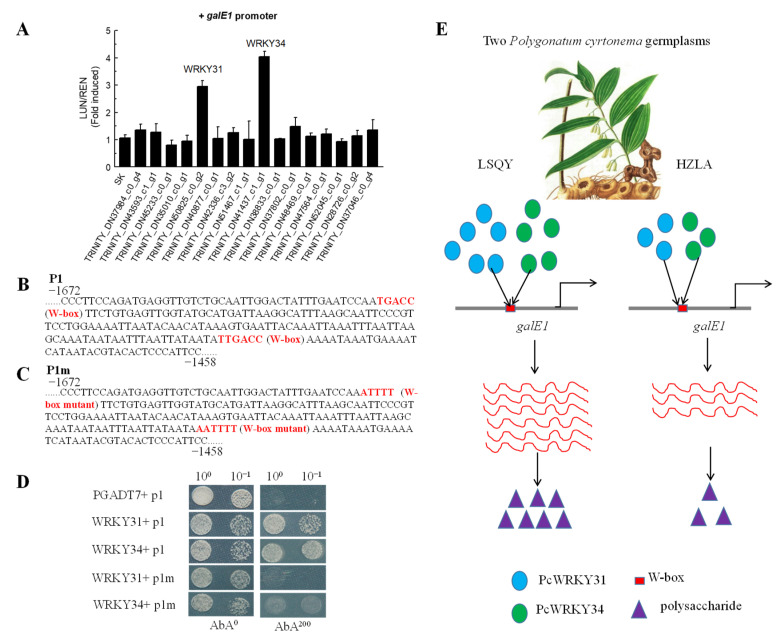
Identification of transcription factors regulating *PcgalE1*, a key polysaccharide biosynthesis gene, by dual-luciferase and Y1H assays. (**A**) Identification of transcription factors activating the promoter of *PcgalE1* by dual-luciferase assay. The LUC/REN value of the promoter’s empty vector was set as 1 as a calibrator. Each value represents the mean ± SD of three independent experiments. (**B**) The bait fragments used to construct the reporter vectors in the Y1H assay. (**C**) The bait fragments with W-box mutants used to construct the reporter vectors in the Y1H assay. (**D**) Interaction of WRKY31 and WRKY34 with a promoter fragment of *PcgalE1* and its mutated sequence in Y1H assays. Yeast was cultured on Leu-lacking SD medium with or without 200 ng mL ^−1^ of Aureobasidin A (AbA) at 30 °C for 72 h. (**E**) A hypothetical model of PCP biosynthesis regulated by PcWRKY31 and PcWRKY34 in two *Polygonatum cyrtonema* germplasms.

In order to identify transcription factors involved in modulating *PcgalE1* expression, all the transcription factors with full-length CDS were selected from the DEGs between LSQY and HZLA. These transcription factors include WRKY, MYB, NAC, ERF, MADS-box, and other family transcription factors (Table S5). In addition, the 1.85 kb length of the *galE1* promoter fragment was also isolated by the genome walking method from genomic DNA (Figure S2). Then, a dual-luciferase assay was performed to detect the effect of transcription factors on the activation of *PcgalE1* expression. We found that transcription factors, TRINITY_DN50825_c0_g2 and TRINITY_DN41437_c1_g1, annotated as PcWRKY31 and PcWRKY34, respectively, that could activate the expression of *PcgalE1* (Figure 5A). Other transcription factors could not activate the expression of *PcgalE1* (Figure 5A).

WRKY proteins bind to W-boxes containing the core sequence (T)(T)TGAC(C/T) of target gene promoters [44]. We found two W-boxes in the 1.85 kb length of the *PcgalE1* promoter sequence. Therefore, we speculated that PcWRKY31 and PcWRKY34 might directly bind to these two W-boxes. To verify this hypothesis, the Y1H method was used. We used a fragment of about 210 bp surrounding the two W-boxes (Figure 5B) and the same fragment with the mutant W-boxes (Figure 5C) as baits for the Y1H assay. We found that yeast co-transformed with PcWRKY31 or PcWRKY34 and the natural promoter region but not with PcWRKY31 or PcWRKY34 and the corresponding mutant fragment, which grew well in the selective media (Figure 5D). These findings indicated that PcWRKY31 and PcWRKY34 could bind to the W-boxes of the *PcgalE1* promoter to activate AUR1-C expression. Based on our results, we proposed a new hypothetical PCP biosynthesis model regulated by PcWRKY31 and PcWRKY34 (Figure 5E). Some signals may induce a relatively higher expression of PcWRKY31 and PcWRKY34 in LSQY and a relatively lower expression of PcWRKY31 and PcWRKY34 in HZLA. PcWRKY31 and PcWRKY34 could further upregulate the *PcgalE1* expression by binding to the W-box in the *PcgalE1* promoter. As a result, the PCP content in LSQY increased more than in HZLA.

## 3. Discussion

*Polygonatum* Mill. is a member of the *Liliaceae* family and is widely distributed in southwest China [45]. The flowers and leaves of this genus are of ornamental value, and the tubers in the underground have important medicinal and edible values [46]. According to modern research, *Polygonatum* is rich in steroidal saponins, flavonoids, PCPs, and other active substances, which have anti-inflammatory, hypolipidemic, anti-tumor, analgesic, hemostatic, and immune regulation effects [2,46,47,48]. The preparation and content determination of *Polygonatum* PCPs have been reported [49]. Owing to differences in growth habitats, *Polygonatum* PCP contents produced in different regions vary from 10 to 20% [49]. Here, we analyzed the PCP content of *Polygonatum* collected from different regions of Zhejiang province. In agreement with a previous report, we found that the PCP content in *Polygonatum* fluctuated between 7.18 and 11.84%. Therefore, conducting transcriptome sequencing comparing two regional species to the highest and lowest PCPs can help unveil the synthesis mechanism of PCPs.

RNA sequencing has been the most effective approach for analyzing functional genes and accurately quantifying their expression without a reference genome [50]. Dozens of medicinal plants have been analyzed by RNA-Seq so far [49]. The relationship between active substances and gene expression was confirmed by transcriptome analysis of *Lonicera japonica* [51]. A transcriptome study of *Glycyrrhiza uralensis* found 16 genes related to the synthesis of glycyrrhizic acid backbone. Through quantitative verification, nine possibly related genes were discovered, further deepening the biological understanding of glycyrrhizic acid synthesis [52]. Five genes were identified in ginsenoside synthesis, including UDP glycosyltransferase and cytochrome P450 [53]. RNA sequencing was used to explore the genes of flavonoid and PCP biosynthesis pathways in *Abelmoschus esculentus* [50]. In our study, two types of non-model plant *P. cyrtonema* were used for RNA-Seq analysis. A total of 4514 Gb of sequence data consisting of 150,464,765 short reads were generated by RNA-Seq. Our sequencing produced 108,195 unigenes (N50:1100bp) with a mean length of 855.15 bp after assembly, facilitating the investigation of PCP biosynthesis on a molecular level.

Present research regarding the biosynthetic pathway of PCPs mainly focuses on fungi [54]. Research on plant PCPs begins with Arabidopsis cell wall PCPs, and the pathway related to PCP metabolism gradually becomes clear with the research of key enzymes [55]. The rapid development of high-throughput sequencing technology makes studying PCP pathways more convenient [50]. For instance, Gao et al. [56] analyzed and verified the key genes in the PCP biosynthesis pathway of *Codonopsis pilosula* by transcriptome analysis, which was the first report on PCP synthesis pathways in medicinal plants. Their research showed that *Codonopsis pilosula* PCP mainly comprises glucose, rhamnose, arabinose, galactose, xylose, and mannose. Although there are many kinds and different PCP structures in plants, the PCP precursor synthesis pathway and monosaccharide repeat units are basically the same [36]. HK and FRK are the key enzymes for PCP synthesis, catalyzing fructose to fru-6p [33]. The isolation and expression characteristics of the HK gene in tea under various abiotic stresses showed that HK and FRK were the key enzymes during plant growth and development [57]. In this study, we collected *P. cyrtonema* germplasm from different regions, analyzed PCP content, and used transcriptome methods to identify several key genes for PCP synthesis. We identified a PCP biosynthesis pathway gene, *PcgalE1,* that has the most significant difference in its expression between LSQY and HZLA. The result suggests that *PcgalE1* may contribute to the difference in PCP biosynthesis between LSQY and HZLA. The overexpression of *PcgalE1* consistently resulted in more PCP accumulation. These findings indicate that the key genes in PCP synthesis or those leading to diverse PCP content in different germplasm resources vary among different species.

Previous studies demonstrated that some transcription factors play diverse roles in modulating PCP biosynthesis. The mutation of an R2R3-MYB gene, MYB61, in Arabidopsis significantly reduced the PCP content of seed mucilage [39]. The overexpression of DoMYB75 significantly increased the water-soluble PCPs in Arabidopsis [58]. However, these reports did not demonstrate the molecular regulatory mechanism of these transcription factors in PCP biosynthesis. Recently, some WRKY genes have proven to play important roles in secondary metabolism. The first regulator, *CjWRKY1*, was identified in the biosynthesis of berberine from *Coptis japonica* Makino. The transient expression of *CjWRKY1* in *Coptis japonica* protoplasts upregulated the expression level of berberine biosynthetic genes [59]. However, these reports did not demonstrate the molecular regulatory mechanism of these WRKYs in PCP biosynthesis. Here, two WRKY transcription factors, PcWRKY31 and PcWRKY34, were identified from the DEGs analysis and positively regulated *PcgalE1* expression by the dual-luciferase assay and Y1H method, suggesting that these two transcription factors may act as new candidate regulators for PCP biosynthesis in plants.

## 4. Materials and Methods

### 4.1. Plant Materials

Different *P. cyrtonema* germplasms were collected from 11 wild populations in Zhejiang province in November 2018. The germplasms were first recognized by the corresponding author Zhengming Tao who is major in plant taxonomy. Subsequently, Renyong Hu, an expert in the field of plant taxonomy from Wenzhou University, conducted a recognition of the species again. These *P. cyrtonema* germplasms were then cultivated in an experimental field plot attached to the soil of the gathering areas at Zhejiang Institute of Subtropical Crops, China. In November 2019, 2–3-year-old sections of rhizomes from 11 different provenances were isolated to detect the PCP content. We then conducted the transcriptomes of rhizomes from two germplasms with the largest contrast in PCP content.

### 4.2. Isolation and Detection of Total PCPs

Previously, we conducted an analysis of polysaccharide content from *P. cyrtonema* with 1–5-year-old rhizomes and found that the highest polysaccharide content was present in the 2–3-year-old rhizomes (Table S6). As a result, we opted to assess the polysaccharide content in the 2–3-year-old rhizomes of different *P. cyrtonema* germplasms. Clean samples from 2–3-year-old sections of rhizomes were dried in the oven at 60 °C till constant mass (20 h). Three biological replicates were used for each sample. The samples were ground into powder (0.25 g) and mixed with 20 mL of 80% ethanol (mass: solvent ratio is 1:80), followed by ultrasonic extraction for 1 h in pure deionized water at 85 °C (repeated 3 times). After immediate filtration, the precipitate was then dissolved in a 250 mL volumetric flask filled with water. The mixture was chilled to room temperature, and water was added at a constant volume. The PCP content was determined by the anthrone–sulphuric acid method (standard curve of glucose at 582 nm: y = 1.1061x − 0.0017, R^2^ = 0.9991).

### 4.3. Illumina Sequencing and Analysis

Two germplasms with the largest contrast in PCP content were selected from the 11 wild *P. cyrtonema* germplasms to conduct the transcriptome analysis. About 2–3-year-old rhizomes were collected from 3 plants for each biological replicate. Three biological replicates were used for each sample. mRNA was extracted from total RNA, and then cDNA libraries were constructed and sequenced by the Illumina HiSeqTM 2000 system. The raw data were deposited into the BioProject database of NCBI (accession number PRJNA874467). The CDSs of unigenes were predicted using TransDecoder. RPKM (reads per kb per million reads) was used to determine the expression level of unigenes. The unigenes were annotated by BLASTX against the databases of Nr, Swiss-Prot, KEGG, KOG, GO, and trEMBL. Differential expression genes (DEGs) were analyzed by the DESeq R package (1.10.1). Genes with an adjusted *p*-value < 0.01 and absolute value of log_2_ (Fold change) > 1 found by DESeq were assigned as differentially expressed. The clusterProfiler R package and KOBAS software (v2.0.12) analyzed the GO enrichment and KEGG pathways, respectively.

### 4.4. qRT-PCR Analysis

DEGs from the PCP biosynthesis pathway were selected for further testing by qRT-PCR. Total RNA was isolated from two germplasm rhizomes with the largest PCP content contrast and reverse transcribed to cDNA with HiScript^®^ II Q RT Super Mix (+gDNA wiper) (Vazyme Biotech, Nanjing, China). We used a C1000 Touch™ Thermal Cycler system (Bio-Rad, Hercules, CA, USA) and SYBR Premix Ex Taq to perform qRT-PCR. We selected a *P. cyrtonema* ACTIN to serve as the inner reference. All primers are listed in Table S7. The 2^−ΔΔCt^ method was used to calculate gene expression. Three biological replicates were used for each sample.

### 4.5. Promoter Sequence Cloning

Genome Walker Universal Kit (Clontech, Mountain View, CA, USA) was used to clone the *galE1* promoter. Briefly, genome walker libraries were constructed according to the user manual. The inner and outer aptamer primers and two galE1 gene-specific primers were used to perform nested PCR. The *galE1* promoter fragment of about 1.85 kb was amplified from the genomic DNA.

### 4.6. Transient Expression of galE1 in Tobacco Leaves

The CDS of *galE1* without stop codon was cloned in-frame in front of the GFP coding region in the binary vector *35S::GFP* (modified from pCAMBIA1300), thus placing *galE1-GFP* under the control of the *35S* promoter. The primers are listed in Table S7. The resultant *35S::galE1::GFP* construct was introduced to the *Agrobacterium tumefaciens* GV3101 strain. The positive clones were transiently expressed in *Nicotina benthamiana* leaves. The GFP signal was detected by confocal laser scanning microscopy (LSM510: Karl Zeiss, Jena, Germany).

Samples used for total PCP determination were collected from leaves after transient expression for 5 d and from wild-type tobacco leaves. About 0.8 g of sample was ground with liquid nitrogen for the total PCP determination according to the method described above. Three biological replicates were used.

### 4.7. Dual-Luciferase Assay

A transient dual-luciferase assay was used to detect the transactivation activity of transcription factors to the *galE1* promoter. Each of the transcription factors was cloned into the pGreenII 62-SK effector vector. The *galE1* promoter was introduced to the pGreenII 0800-LUC dual-reporter vector. The reporter and effector vectors were then co-expressed in tobacco leaves at a ratio of 1:9 (reporter: effector). Table S7 shows the primers. LUC- and REN-luciferase activities were detected using the dual-luciferase assay kit (Promega) on the Luminoskan Ascent Microplate Luminometer (Thermo Fisher Scientific, Waltham, MA, USA). The results were calculated by the ratio of LUC to REN.

### 4.8. Yeast One-Hybrid Assay (Y1H)

The CDSs of *PcWRKY31* and *PcWRKY34* were fused into the pGADT7 vector, respectively. The *PcgalE1* promoter fragments were fused into the pAbAi vector. Plasmids were transformed into the Y1H gold yeast strain and cultured on Leu-lacking SD medium with or without 200 ng mL ^−1^ AbA at 30 °C for 72 h.

### 4.9. Statistical and Sequence Analysis

Statistical analyses were carried out using SPSS version 16.0 (SPSS Inc., Chicago, IL, USA). Significant differences of PCP contents and relative expression of genes analyzed by qRT-PCR between two groups were determined using one-way ANOVA followed by Tukey’s test at *p* < 0.05. The statistics of F and degree of freedom are shown in Table S8. Amino acid sequence alignment was performed using DNAMAN software (version 9) with default parameters (gap penalty was set at 3, K-tuple at 1, and number of top diagonals at 5).

## 5. Conclusions

In conclusion, comparative transcriptome analysis was performed on two germplasms with the largest contrast in PCP content. We identified 654 DEGs, important genetic resources, in our research. We proposed the PCP biosynthetic pathway in *P. cyrtonema* and identified the genes involved in PCP biosynthesis from the transcriptome data. In *P. cyrtonema*, PMM, gmd, and galE may play key roles in PCP accumulation, which differ from those in other plants. Furthermore, overexpression of *PcgalE1* in tobacco leaves increased PCP levels. PcWRKY31 and PcWRKY34 were identified, for the first time, to regulate PCP biosynthesis by activating the transcription of *PcgalE1.* Our study preliminarily revealed the unique molecular mechanism of the PCP biosynthesis pathway in *P. cyrtonema*. Our findings have laid a theoretical foundation for a new breeding variety, the rational development and utilization of *P. cyrtonema,* and sustainable development of the *P. cyrtonema* industry.

## Figures and Tables

**Table 1:** Determination of polysaccharide contents in different *Polygonatum cyrtonema* germplasms in Zhejiang Province. Different letters indicate significant differences between samples at *p* < 0.05. Data are means ± SD of three biological replicates.

No.	Germplasms	Longitude (E)	Latitude (N)	Altitude (m)	Polysaccharide Content/%
1	Wenzhou City, Taishun country (WZTS)	119.69	27.65	773	9.08 ± 0.84 bcd
2	Wenzhou City, yongjia country (WZYJ)	120.76	28.51	724	8.14 ± 0.28 de
3	Wenzhou City, Yueqing country (WZYQ)	121.06	28.39	88	8.33 ± 0.77 cde
4	Wenzhou City, Rui’an country (WZRA)	120.34	27.83	268	7.35 ± 0.46 e
5	Lishui City, Longquan country (LSLQ)	119.24	28.04	589	9.74 ± 0.46 b
6	Lishui City, Qingyuan country (LSQY)	118.99	27.60	352	11.84 ± 1.18 a
7	Jinhua City, Pan’an country (JHPA)	120.55	28.99	574	9.58 ± 0.56 b
8	Taizhou City, Tiantai country (TZTT)	121.01	29.20	177	9.51 ± 0.46 bc
9	Hangzhou City, Chun’an country (HZCA)	118.95	29.49	117	9.18 ± 0.52 bcd
10	Hangzhou City, Lin’an country (HZLA)	119.45	30.32	436	7.18 ± 0.25 e
11	Quzhou City, Jiangshan country (QZJS)	118.73	28.83	89	8.68 ± 0.80 bcd

## Data Availability

The transcriptional data were deposited into the BioProject database of NCBI (accession number: PRJNA874467).

## Supplementary Materials

The supporting information can be downloaded at: https://www.mdpi.com/article/10.3390/ijms241612943/s1.
