# Supplementary material for: Comparative Transcriptome Profiling Reveals Two WRKY Transcription Factors Positively Regulating Polysaccharide Biosynthesis in Polygonatum cyrtonema
Source: Int J Mol Sci. 2023 Aug 18;24(16):12943. doi: 10.3390/ijms241612943 (PMC10454705; doi:10.3390/ijms241612943)
Supplement: Supplementary file 1 [file ijms-24-12943-s001.zip › ijms-2527980-supplementary.pdf]

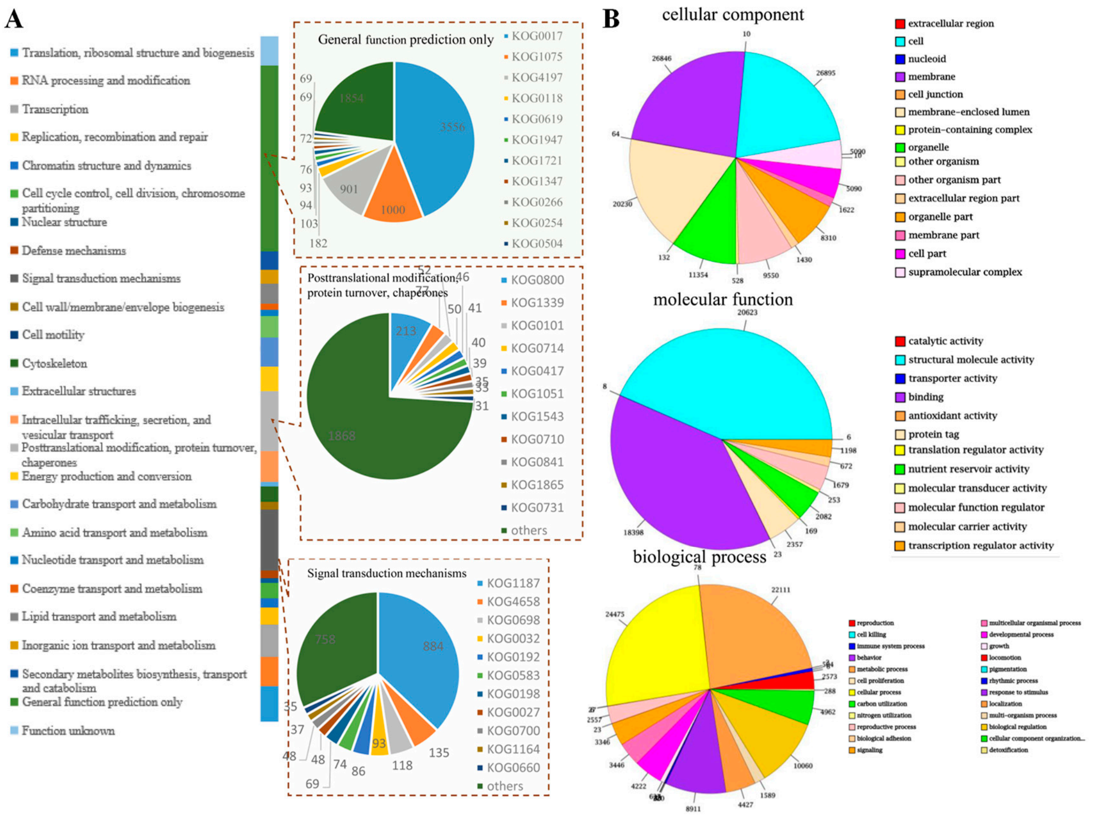

Fig. S1. Function classification of all unigenes (A) KOG function classification of all unigenes. (B) GO function classification results of the annotated unigenes in *Polygonatum cyrtonema*.

CCGGAGATTGGAATATCCAAAATACAGTACACATCATCAAGGGTAATGGT 50

CATCTCACCAAAAGGTAAATGGAATGTATTAGTCTCCGGCTGCCACCTCT 100

CAACAAATGCTGAGACTACTACCTTATTGGCATACCTATAGGAACAATCA 150

ATAAGGCTCATCAAACCGGATCTCTGAATATAACCCTTCCAGATGAGGTT 200

GTCTGCAATTGGACTATTTGAATCCAA**TGACC**ATTCTGTGAGTTGGTATG 250

CATGATTAAGGCATTTAAGCAATTCCCGTTCCTGGAAAATTAATACAACA 300

TAAAGTGAATTACAAATTAAATTTAATTAAGCAAATAATAATTTAATTAT 350

AATA**TTGACC**AAAATAAATGAAAATCATAATACGTACACTCCCATTCCAA 400

ATCTCTGAGGCAATATGTGTCTTGAAACTTCGTAAAATCCTAGGGTCCTG 450

TGGCCCACCGGGAAATGGTCCTGTCAACTCCTCATGCTCAGATTCAACAG 500

CCTGCGCTTCTACATTCGCTTCTACATTCAGATGCTCCTCTTCTTGAACG 550

TTTGGTTGTATAACTGGTTCATCCTGTACAACTTGTTCTGTCTCTAACTC 600

TGATATATGAACTCGTCTTGCTGCATCTGCTGCACGTCGGGCTGATGCAG 650

TCGTTCTCTTTGTCCTTCGAGATGTGCCAGCCTCATCTACAAATTAAATT 700

AAGATAGTACTGTCAAAAAAATATGATAAATGACTATACAAAATAAGCAT 750

ACCATGACTTCATATATTATAATTTTAGTAAACAAAAACAAATATTGTAC 800

AGTAGAACAAGAAAAAAAAATACACATTGAATATTGTGAAAACTATTTAT 850

GTAGTAAGAAAAGGGAATAGAAGGAAATGAGTTGGGAGTTCATTAGAAAT 900

TGATAGAGAAAGTTCAGTATCTCTACAGAACGAGAGAGAGTATGAGAAGC 950

TAGCAATGATCATGTGTGACACTGATCATTGCATCATTCAAAAACACAAC 1000

TGATCAATGTATATATAGCATAGAGAGAGAGAGAAAACAAAGTGGTAA 1050

TGTAATGTGAATGTGACTGTGCTAGTAAGAGTATAGTGATGAGCTCAAGT 1100

CAGCTAGCAGCAATATACAAGCAGTCAGTCGTCGGCCTAAAGAAACCGAC 1150

CAAATAGTCTTTCGTCGGCCTATTTAGGCCGACAGATTAATATTTGGTCG 1200

GTCTTAACAGGCCGACCAAATAGTAATCTGTCGGCCTAAATAGACCGACA 1250

AAAATTAGCCAAGTCGGGTTCTCGGGGCCGACCGACTGTCGCATGAACAA 1300

ACGTTACAGAAAGTTCAAAAAACTTTAATTTTATGATTTATAATCTTAAA 1350

ACAATACCTTGATTGGTTCGAGAAGACATCTAAAGCGTTTGATAGATTCT 1400

CAAAATGAATATATCCTGAATCAAATTAAAAAAACAATCAGTTTAACCCA 1450

AATTTTTTTCTCCAACTAAATAAAAATGAAAGAAATCGAATTAGGGTTTT 1500

CTATTAGGTACAAAGCTATAAAAACTTACCTAAATCCTTGTCTTCTTCTC 1550

TACTCCTCCTACTCTTCTTCACGATCGGCTCCTCCTCCTCTTCTTCGCGA 1600

TGGTTGTTCCTCTTCTTCTTCGCGTTTTTTTTTTTTTTTTTTTACAGTCA 1650

AGTTAATTTTTTCTACTTGGTTGACTGGGTTTACTGAGCAAATCAAATGG 1700

GTATTTTTGGTATAGATAAAGGGTAAACTGAGCAAAATGTTGGGTTTGTT 1750

TTGGTTATGGGTTTACTGAGCAAAAAATTGGGTGCCAATAGAATTCCCCA 1800

ATGAGTTTTGAATGGGGCTACTGAGCAATTTTGGGGGTTCAAATAGAATT 1850

ACCCT**ATG** 1855

↓

Translation Start Codon

Fig. S2. The sequence and cis elements analysis of *PcgalE1* promoter. The position of nucleotide in the promoter is opposite to the translational start site (ATG) that was marked as +1. W-box cis elements are highlighted as red color.
